# Phosphatase to kinase switch of a critical enzyme contributes to timing of cell differentiation

**DOI:** 10.1128/mbio.02125-23

**Published:** 2023-12-06

**Authors:** Trisha N. Chong, Mayura Panjalingam, Saumya Saurabh, Lucy Shapiro

**Affiliations:** 1 Department of Developmental Biology, Stanford University School of Medicine, Stanford, California, USA; 2 Department of Chemistry, New York University, New York, New York, USA; Carnegie Mellon University, Pittsburgh, Pennsylvania, USA

**Keywords:** *Caulobacter*, cell differentiation, histidine kinase, phosphatase, two-component systems, PAS domain

## Abstract

Cell differentiation is an essential biological process that is subject to strict temporal regulation. *Caulobacter crescentus* undergoes obligate differentiation from a swarmer cell to a stationary, replication-competent stalked cell with each cell cycle. We report that the switch from phosphatase to kinase activity of the histidine kinase PleC contributes to timing this differentiation event. PleC Per-Arnt-Sim (PAS) domain interaction with the polar scaffold protein PodJ localizes PleC to the cell pole and inhibits *in vivo* kinase activity. Upon PodJ degradation, released PleC switches to its kinase form and phosphorylates the PleD diguanylate cyclase, initiating the signaling pathway responsible for differentiation. While PodJ inhibits PleC kinase activity, it does not impact PleC phosphatase activity on DivK, which is required for pili biogenesis and flagellar rotation. Thus, PleC PAS domains affect enzymatic function on diverse substrates by relying on context-dependent binding partners, thereby controlling the timing of *Caulobacter* cell differentiation.

**IMPORTANCE:**

The process of cell differentiation is highly regulated in both prokaryotic and eukaryotic organisms. The aquatic bacterium, *Caulobacter crescentus*, undergoes programmed cell differentiation from a motile swarmer cell to a stationary stalked cell with each cell cycle. This critical event is regulated at multiple levels. Kinase activity of the bifunctional enzyme, PleC, is limited to a brief period when it initiates the molecular signaling cascade that results in cell differentiation. Conversely, PleC phosphatase activity is required for pili formation and flagellar rotation. We show that PleC is localized to the flagellar pole by the scaffold protein, PodJ, which is known to suppress PleC kinase activity *in vitro*. PleC mutants that are unable to bind PodJ have increased kinase activity *in vivo*, resulting in premature differentiation. We propose a model in which PodJ regulation of PleC’s enzymatic activity contributes to the robust timing of cell differentiation during the *Caulobacter* cell cycle.

## INTRODUCTION

**Fig 1:**
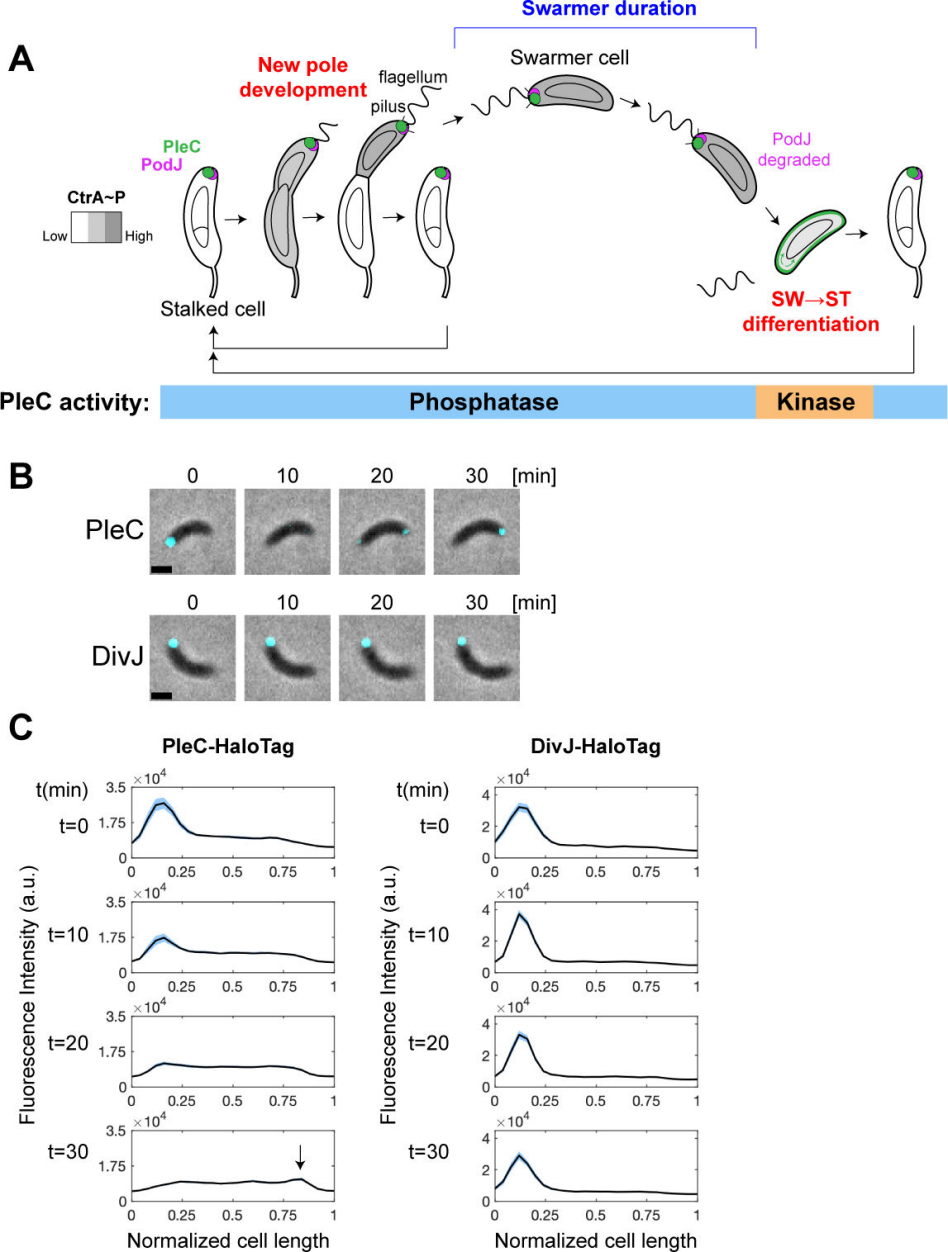
PleC molecules exhibit polar translocation during differentiation. (A) Schematic of PleC, PodJ, and CtrA~P localization throughout the cell cycle and the corresponding PleC enzymatic state resulting from PodJ inhibition of PleC kinase activity at the cell pole. (B) Representative merged phase and fluorescence time-lapse images of PleC-HaloTag (top) and DivJ-HaloTag (bottom) molecules pulse labeled with 2 nM JF549 dye for 10 minutes. For PleC-HaloTag, cells were labeled immediately after synchrony. For DivJ-HaloTag, swarmer cells were allowed to progress to stalked cells for 40 minutes prior to labeling. Scale bar = 1 µm. (C) Fluorescence intensity profiles along normalized cell length axes of PleC-HaloTag (*n* = 47) and DivJ-HaloTag (*n* = 52) at 10-minute time points. Light blue shaded area denotes the standard error of the mean. Arrow points to peak in fluorescence at the incipient flagellar pole.

Cell differentiation is the process by which a cell acquires a new cell fate that makes it specifically suited for its environmental or developmental context. The process of cell differentiation can be triggered by either an external cue or an internal signaling circuit. The aquatic bacterium, *Caulobacter crescentus*, has a di-morphic cell cycle (Fig. 1A) in which a swarmer cell differentiates into a sessile, replication-competent stalked cell in response to both external signals and internal regulatory pathways (1, 2).

Cell division in *Caulobacter* yields a motile swarmer cell and a stationary stalked cell which immediately initiates DNA replication. The swarmer daughter cell must differentiate into a stalked cell before undergoing replication. Swarmer to stalked cell differentiation involves shedding the polar flagellum, proteolysis of the chemotaxis machinery, pili retraction, DNA replication initiation, and stalk biogenesis (2). A critical component of this differentiation event is the histidine kinase, PleC, which acts as either a kinase or a phosphatase at distinct times in the cell cycle. To initiate cell differentiation, the diguanylate cyclase, PleD, must be phosphorylated in order to synthesize the second messenger, cyclic-di-GMP (c-di-GMP) (3). C-di-GMP directly binds to a ClpXP protease adaptor protein, activating proteolysis of the master transcription factor CtrA and enabling swarmer to stalked cell differentiation (see Fig. 3A) (4). It has been previously reported that both PleC and the histidine kinase, DivJ, can act as a kinase on PleD *in vitro* (5). Conversely, it has been shown both *in vitro* and *in vivo* that PleC acts as a phosphatase on its other cognate response regulator, DivK (5, 6). PleC phosphatase activity on DivK in pre-divisional cells leads to downstream phosphorylation and activation of newly synthesized CtrA, which is required for the development of the new pole, including flagellar rotation and pili biogenesis (see Fig. 6A) (7, 8). Mutants lacking PleC phosphatase activity are immotile and unable to form pili. This phenotype can be rescued by restoring specifically PleC phosphatase and not kinase activity (7).

While the signaling pathway that governs swarmer to stalked cell differentiation has been extensively characterized, the mechanisms responsible for timing differentiation are poorly understood. Multiple reports have shown that interaction with a surface can trigger swarmer to stalked cell differentiation, resulting in a shortened swarmer cell duration. This is facilitated by signal transduction through either the polar pili or the flagellum (9–13). However, when grown in nutrient-rich liquid media, isolated swarmer cells reliably undergo differentiation into stalked cells after approximately 40 minutes (14). This implies that in the absence of surface stimulation, an internal molecular circuit controls the timing of cell differentiation. Paul et al. previously showed that PleC phosphorylates PleD *in vitro* and that addition of DivK promoted this phosphorylation reaction. Based on these *in vitro* experiments, the authors proposed that allosteric binding of DivK to PleC at the incipient stalked pole switches PleC to its kinase conformation, enabling robust phosphorylation of PleD, which in turn contributes to the swarmer to stalked cell differentiation (5).

Here, we provide evidence that PleC interaction with the polar scaffold protein PodJ recruits PleC to the cell pole and maintains PleC in its phosphatase conformation when the two proteins colocalize at the cell pole (Fig. 1A) (15–17). Prior to cell differentiation, PodJ is cleaved from the membrane and fully proteolyzed (18), releasing PleC from the cell pole and enabling its kinase activity. PleC kinase phosphorylates PleD, initiating the molecular pathway that results in swarmer to stalked cell differentiation (see Fig. 3A). After differentiation, diffuse PleC is captured by newly synthesized PodJ at the pole opposite the stalk, reverting PleC back to its phosphatase form (Fig. 1A). We show that a mutant lacking PleC PAS (Per-Arnt-Sim) domains, whose kinase activity is not inhibited by PodJ, prematurely degrades CtrA and initiates the early onset of chromosome replication. Notably, only PleC kinase activity on PleD and not its phosphatase activity on DivK is affected by PodJ inhibition. In this way, PodJ proteolysis enables a PleC phosphatase to kinase switch and initiates the molecular pathway that results in swarmer to stalked cell differentiation.

## RESULTS

### PleC molecules exhibit polar translocation during differentiation

PleC localizes to the flagellar pole throughout most of the cell cycle except for a brief period during swarmer to stalked cell differentiation, when PleC is observed to vacate the old flagellar pole and appear at the opposite, incipient flagellar pole (Fig. 1A) (6, 7). To determine whether PleC appearing at the incipient flagellar pole is composed of only newly synthesized PleC molecules or those that are released from the old flagellar pole, we performed a pulse labeling experiment on cells with PleC C-terminally fused to HaloTag (19) as the sole copy of PleC encoded at the endogenous *pleC* locus. We pulse labeled synchronized swarmer cells with the fluorescent dye, Janelia Fluor 549 (JF549), that covalently binds to HaloTag. This allowed fluorescent tagging of PleC-HaloTag molecules that were only present at the start of the time-lapse experiment. We observed that pulse-labeled PleC-HaloTag molecules originating from the old flagellar pole appeared as a fluorescent focus at the new flagellar pole (Fig. 1B and C). As a negative control, we observed that pulse-labeled DivJ-HaloTag remained at the stalked pole during stalked cell development into pre-divisional cells (Fig. 1B and C). PleC translocation occurs at the same time in the cell cycle that PodJ is cleaved from the membrane and fully degraded (18). As PodJ is required for PleC polar localization (Fig. S2) (15, 20), release of PleC from the old flagellar pole is likely due to PodJ degradation at that pole. We observed a reduction in the PleC-HaloTag signal when comparing the intensity of the fluorescent focus at the old and new flagellar poles (Fig. 1B and C). This average reduction in peak intensity of 60% was greater than that expected by photobleaching, suggesting that not all PleC-HaloTag molecules translocated to the new pole. This observation is in line with previous reports showing PleC proteolysis at this point in the cell cycle (21, 22). Our results demonstrate that while the majority of PleC molecules are proteolyzed at this point in the cell cycle, a small fraction of PleC molecules evades proteolysis and translocates to the incipient flagellar pole (Fig. 1B and C).

### PleC polar localization is dependent on PAS domains

**Fig 2:**
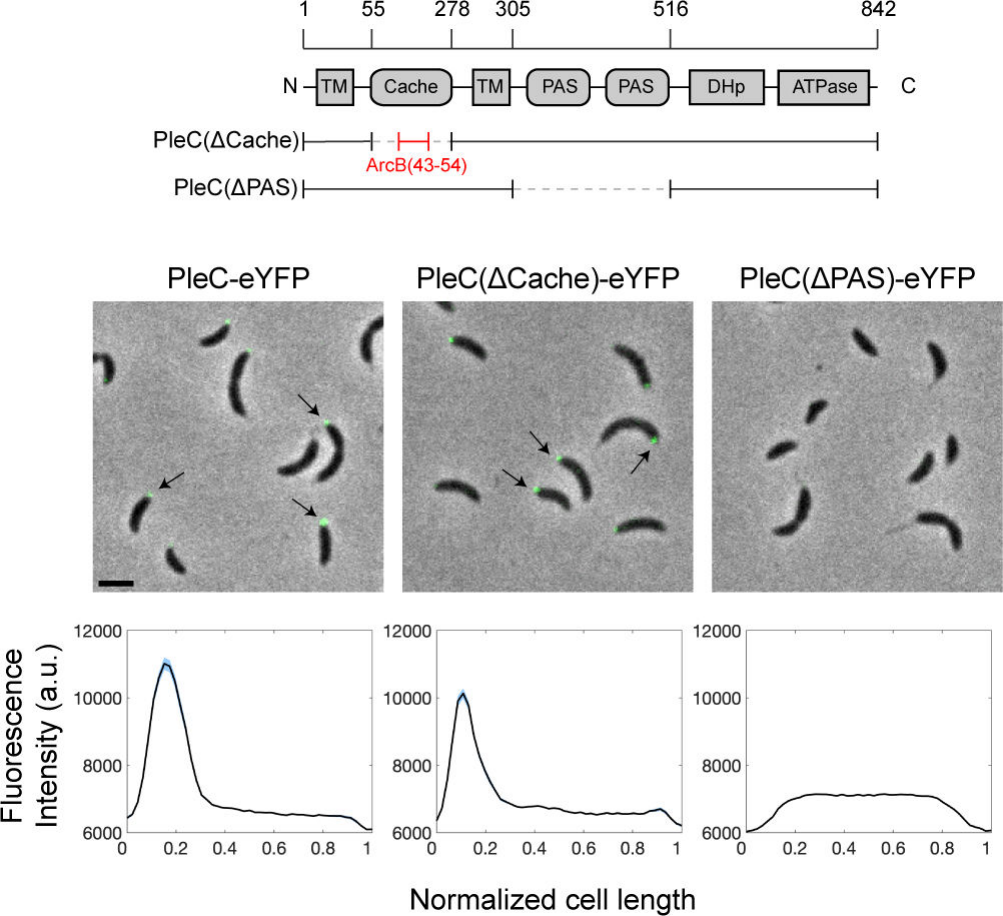
PleC polar localization is dependent on PAS domains. Schematic of PleC domain architecture and PleC domain mutants (top). The *pleC(ΔCache)* variant was generated by replacing the periplasmic Cache domain of PleC with the unstructured periplasmic sequence from the *E. coli* protein ArcB. Representative merged phase and fluorescence images (middle) of PleC domain mutants with C-terminal eYFP. Arrows point to polar fluorescence signals. Scale bar = 2 µm. Line profiles of average fluorescence intensity along normalized cell lengths (bottom). WT *n* = 1,415. *pleC(ΔCache) n* = 1,189. *pleC(ΔPAS) n* = 1,224. Light blue shaded area denotes the standard error of the mean.

PleC polar localization is dependent on the polar scaffold protein, PodJ (Fig. S2) (15, 20). The N-terminus of the PleC multi-domain histidine kinase consists of a periplasmic Cache domain positioned between two transmembrane domains (Fig. 2) (17). Cache domains are periplasmic sensory domains that are homologous to, but structurally distinct from, PAS domains (23). In the cytoplasm, PleC contains two PAS domains, a DHp (Dimerization and Histidine phosphotransfer) domain and a C-terminal ATPase domain (Fig. 2) (17). To identify the domains that are required for PleC polar localization, we constructed two domain deletion mutants: a Cache domain deletion and a PAS domain deletion, both C-terminally fused to enhanced yellow fluorescent protein (eYFP) and expressed as the sole copy of PleC from its native promoter (Fig. 2). The *pleC(ΔCache)* mutant was constructed by replacing the periplasmic Cache domain, PleC(56-278), with the periplasmic linker portion of the *Escherichia coli* protein ArcB(43-54), as this protein should not interact with any proteins specific to *Caulobacter*. The *pleC(ΔPAS)* mutant was constructed by fusing PleC(S305) to PleC(A516), deleting both cytoplasmic PAS domains (Fig. 2).

WT PleC fused to eYFP localized to the flagellar pole of swarmer cells and to the incipient flagellar pole of stalked and pre-divisional cells (Fig. 2; Fig. S2) (6). We observed that the average intensity of the fluorescent polar focus of *pleC(ΔCache)-eYFP* cells was approximately 30% lower compared to Wildtype (WT) cells (Fig. 2). In contrast, PleC(ΔPAS)-eYFP was diffuse in swarmer, stalk, and pre-divisional cells (Fig. 2) (17). We confirmed the stable expression of PleC(ΔCache) and PleC(ΔPAS) by Western blot (Fig. S1). We confirmed that PleC-eYFP is not polarly localized in *ΔpodJ* cells, although PleC is detectable at WT levels by Western blot (Fig. S1 and S2). Together these results suggest that both PodJ and PleC PAS domains are required for PleC polar localization, while the PleC Cache domain is required for WT levels of PleC polar localization (Fig. 2; Fig. S2).

### Loss of PAS domains leads to increased PleC kinase activity

**Fig 3:**
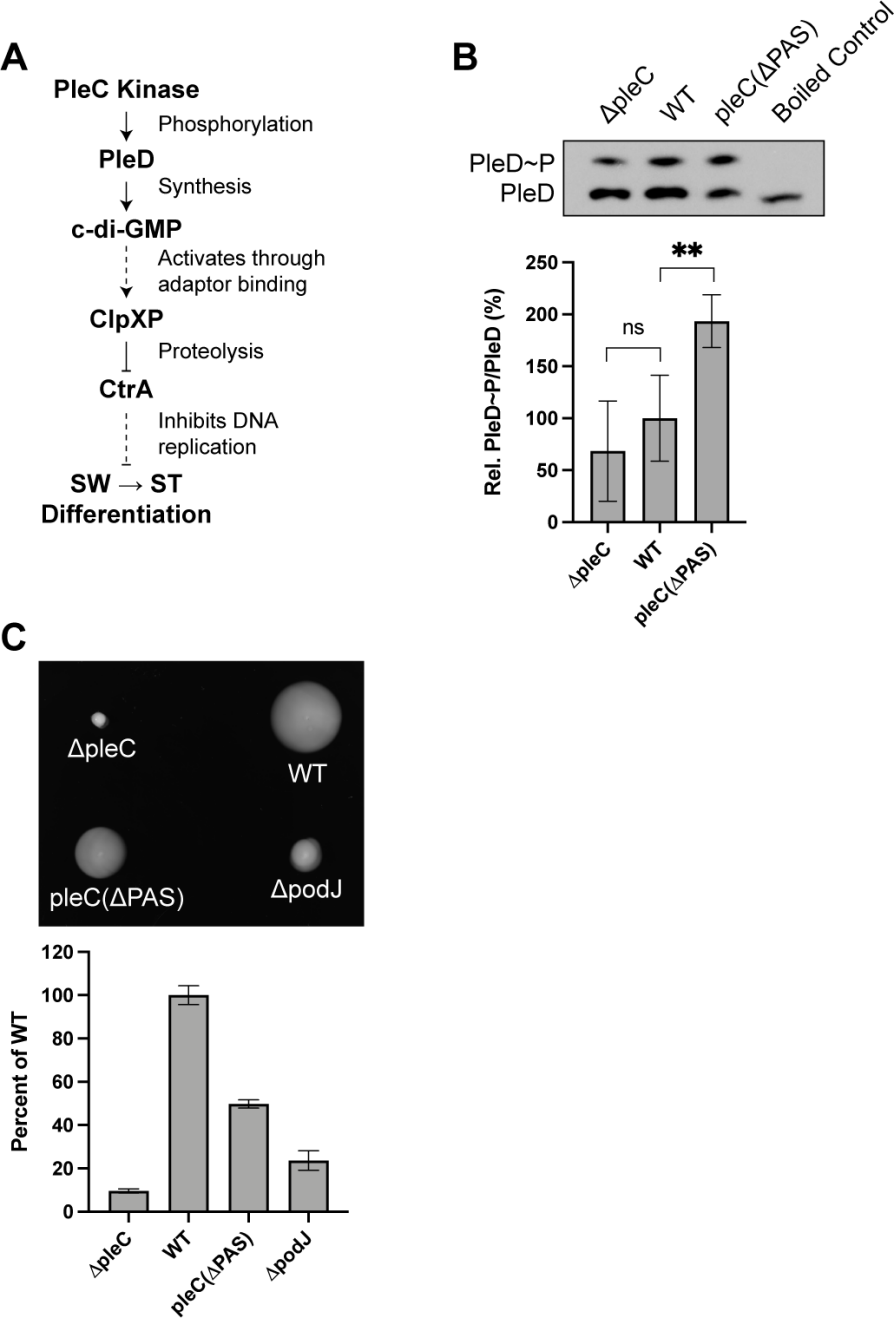
Loss of PAS domains leads to increased PleC kinase activity. (A) The signal transduction pathway from PleC kinase activity on the diguanylate cyclase, PleD, that leads to swarmer (SW) to stalked (ST) cell differentiation. The effects of c-di-GMP on CckA activity are omitted for simplicity but included in Fig. 7A. (B) PhosTag SDS-PAGE immunoblots showing phosphorylated and unphosphorylated PleD-HaloTag-3xFlag using an anti-Flag antibody (top). Bar plot shows the average ratios of phosphorylated to unphosphorylated protein as a percentage of the WT ratio (bottom). Signal density was averaged across four replicates. Error bars indicate standard deviation. An unpaired *t*-test was used to calculate *P* = 0.0085. (C) Swarm plates showing the swarm abilities of mutants in peptone yeast extract 0.3% agar over 3 days (top). Bar plot shows swarm areas as a percentage of WT averaged across 10 replicate plates (bottom). Error bars indicate standard deviation.

The PleC kinase phosphorylates the receiver domain of the diguanylate cyclase PleD (Fig. 3A) (5, 24). To investigate the role of PAS domains on PleC kinase activity *in vivo*, we performed PhosTag gel-electrophoresis to measure the ratio of PleD~P/PleD in cells expressing PleD-HaloTag-3xFlag as the sole copy of PleD expressed from the endogenous locus (25, 26). We found that the ratio of PleD~P/PleD was higher in *pleC*(*ΔPAS)* (193% ± 26% of WT) compared to both WT and *ΔpleC*, suggesting that deletion of PleC’s PAS domains results in increased kinase activity on PleD (Fig. 3B). These results, together with Zhang et al.’s result that PodJ inhibits PleC kinase activity *in vitro*, suggest that the PleC(ΔPAS) mutant protein has increased kinase activity on PleD compared to WT PleC. While PleC has been previously reported to be a kinase of PleD (5), we observed that the ratio of PleD~P/PleD in *ΔpleC* (68% ± 48% of WT) was not significantly lower than that of WT, suggesting that PleC might also act as a phosphatase on PleD, at some point in the cell cycle (Fig. 3B). To obtain the relative levels of the PleD protein, the sum of the signal densities of the phosphorylated and unphosphorylated bands was measured. The estimated total levels of PleD protein were not significantly different when comparing *pleC(ΔPAS)* and *ΔpleC* to WT. Together these results suggest that PAS domains are required for both polar localization and inhibition of PleC kinase activity on PleD (Fig. 2 and 3B).

When phosphorylated, PleD synthesizes the second messenger, c-di-GMP (Fig. 3A) (3). C-di-GMP has been shown to have a role in regulating the lifestyle switch from planktonic to biofilm-forming cells in many bacteria including *E. coli*, *Pseudomonas aeruginosa*, *Salmonella enterica* serovar Typhimurium, and others (27). In *Caulobacter*, c-di-GMP has been previously implicated in reducing motility and stimulating swarmer to stalked cell differentiation (28). To test whether the increased PleD~P/PleD ratio in *pleC(ΔPAS)* coincides with reduced swarm ability, we seeded colonies onto semi-solid peptone yeast extract (PYE) plates and measured the resulting swarm areas. As expected, *ΔpleC* colonies expanded to an area much smaller (10% ± 1% of WT) than that of WT (Fig. 3C) (29). In contrast, the swarm area of *pleC(ΔPAS)* (50% ± 2% of WT) was smaller than that of WT but not as small as that of *ΔpleC* (Fig. 3D). We also observed that the swarm area of *ΔpodJ* was smaller than that of *pleC(ΔPAS)*, suggesting that the *podJ* deletion has other pleiotropic effects related to swarming that are independent of PodJ’s interaction with PleC (Fig. 3D). These pleiotropic effects might be due to insufficient PleD synthesis in *ΔpodJ* (Fig. S4) (30–32). As swarm areas measure the average motility and chemotaxis of a population of cells over the course of a few days, we reasoned that the increased kinase activity of PleC(ΔPAS) on PleD could lead to decreased swarm area through two possible mechanisms: (1) reduced speed of swarmer cells or (2) premature swarmer to stalked cell differentiation, shortening the duration of the swarmer phase of the cell cycle.

### Cells lacking PleC PAS domains swim as fast as WT cells

**Fig 4:**
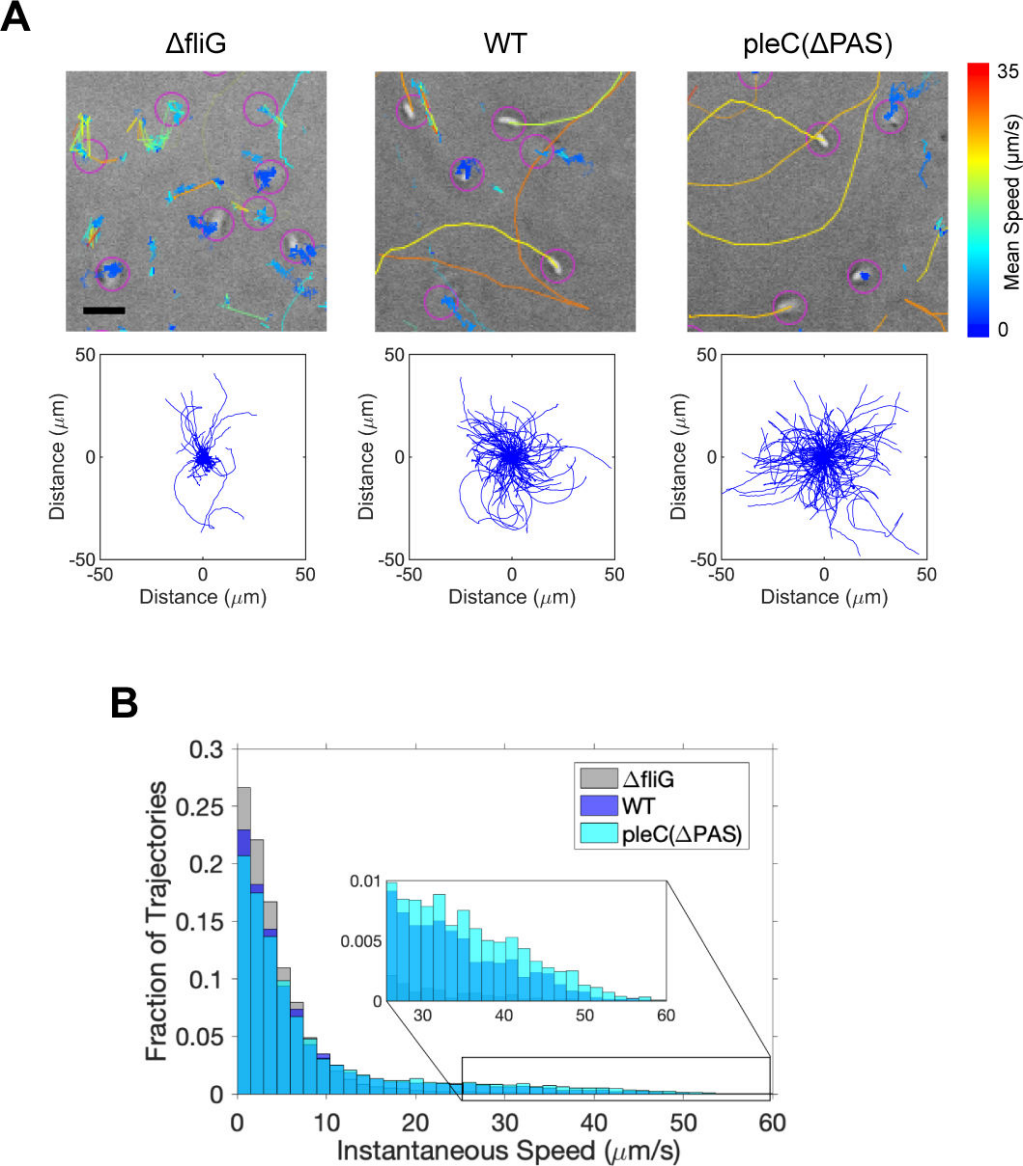
Cells lacking PleC PAS domains swim as fast as WT cells. (A) Representative tracks of individual swarmer cells in M2G with 20% glycerol taken over a time interval of 10.4 seconds (top). Track color corresponds to the track’s mean speed. Scale bar = 4 µm. Plots of trajectories taken over a time interval of 26 seconds with starting time points mapped to coordinate (0,0) (bottom). *ΔfliG n* = 1,524. WT *n* = 1,625. *pleC(ΔPAS) n* = 824. (B) Histogram of the fraction of total trajectories in bins corresponding to instantaneous speed values. Zoomed-in portion of the histogram shows that a larger fraction of *pleC(ΔPAS)* cells have high instantaneous speed values compared to WT cells.

Swarm assays represent a population average of motility and chemotaxis effects. To overcome the limitations of the swarm assay, we quantified motility at the single cell level. Using phase contrast microscopy, we observed the swimming behavior of individual swarmer cells in glass bottom wells. We observed cells at a focal plane above the coverslip as to not capture cells that had settled to the bottom of the well. The majority of WT and *pleC(ΔPAS)* swarmer cells were observed swimming in straight or curved lines, characteristic of *Caulobacter’s* run and flick motility (Fig. 4A) (33). As a negative control, we observed the flagellar mutant, *ΔfliG*, since unlike most *Caulobacter* strains, *ΔpleC* swarmer cells cannot be isolated by density centrifugation (34, 35). In contrast to WT and *pleC(ΔPAS)* swarmer cells, the majority of *ΔfliG* cells exhibited small twitching movements, even when observed away from the coverslip (Fig. 4A).

To gain further insights into the swimming behavior of these cells, we performed segmentation and tracking using the Fiji plugin, TrackMate (36). These analyses allowed for mapping the trajectories of individual swarmer cells and quantification of their instantaneous speeds along those trajectories (Fig. 4A and B). To observe the extent of motility of each strain, we set the starting point of each trajectory to the origin. This analysis showed that *pleC(ΔPAS)* swarmer cells would cover an area similar to that of WT over the same amount of time (Fig. 4A). In addition, the distribution of instantaneous speeds showed that both WT and *pleC(ΔPAS)* had higher fractions of cells that swam at fast instantaneous speeds (>30 µm/s) compared to *ΔfliG* (Fig. 4B). Interestingly, we discovered that a slightly higher fraction of *pleC(ΔPAS)* cells swam at fast instantaneous speeds (>30 µm/s) compared to WT (Fig. 4B). This analysis led us to conclude that the difference in swarm areas between WT and *pleC(ΔPAS)* is not due to differences in the swimming speed of swarmer cells between the two strains. Accordingly, we considered that a shortened swarmer duration of the cell cycle might account for the reduced swarm size of *pleC(ΔPAS)*.

### Loss of PleC PAS domains results in premature swarmer to stalked cell differentiation

Multiple events occur during swarmer to stalked cell differentiation, including dephosphorylation and degradation of the master cell cycle regulator CtrA, initiation of chromosome replication, degradation of the polar chemotaxis machinery, and remodeling of the new stalked pole (4, 37–39). To address the possibility that the reduced swarm size of *pleC(ΔPAS)* is due to a shortened swarmer phase of the cell cycle, we used these developmental events as cell cycle landmarks to determine the difference in swarmer duration between WT and *pleC(ΔPAS)*.

**Fig 5:**
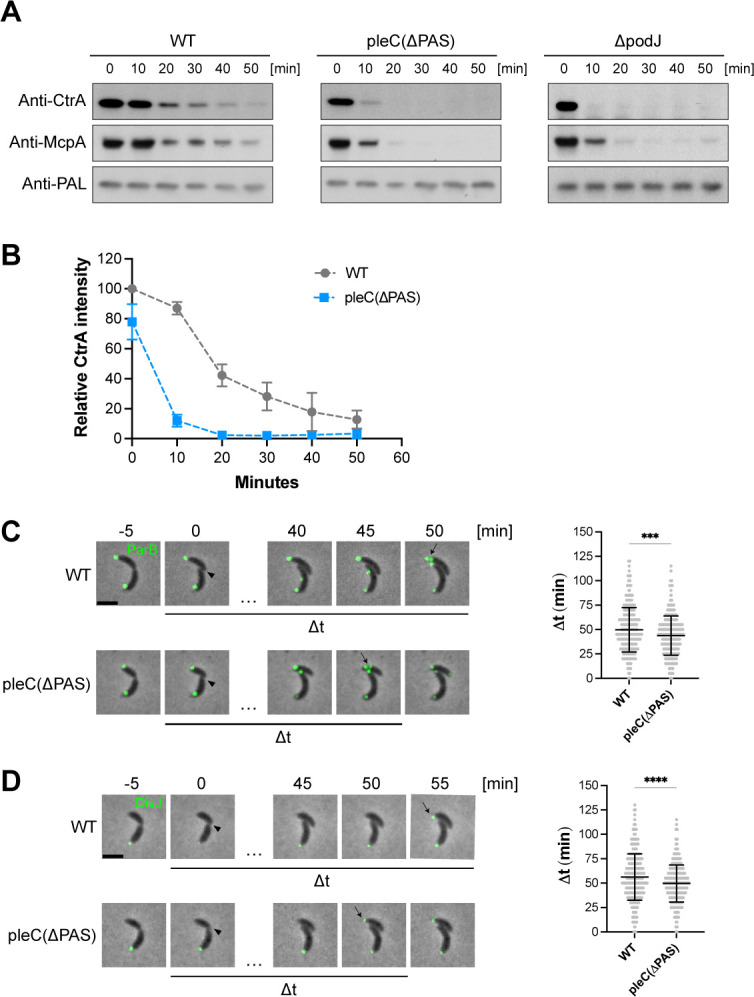
Loss of PleC PAS domains results in premature swarmer to stalked cell differentiation. (A) Western blot showing CtrA and McpA levels of synchronized WT, *pleC(ΔPAS)*, and *ΔpodJ* cells. Western blot against non-cell-cycle-controlled protein PAL shows consistent levels through the cell cycle. (B) Quantification of CtrA signal density averaged across four replicates. WT and *pleC(ΔPAS)* samples were run on the same gel for each replicate. Error bars denote standard deviation. (C) Time-lapse images of eYFP-ParB foci as swarmer cells differentiate into stalked cells and initiate DNA replication. Representative merged phase and fluorescence time-lapse images of WT and *pleC(ΔPAS)* cells expressing *pxyl:eYFP-parB*, induced with 0.01% xylose for one hour. Arrowheads point to cytokinesis events. Arrows point to a single eYFP-parB focus splitting into two foci. Scale bar = 2 µm. Violin plot of Δ*t*, the time interval between cytokinesis and splitting of the eYFP-parB focus into two foci (right). WT *n* = 390. *pleC(ΔPAS) n* = 362. Error bars denote the mean ± standard deviation. An unpaired *t*-test was used to calculate *P* = 0.0002. (D) Time-lapse images of DivJ-eYFP foci as swarmer cells differentiate into stalked cells. Representative merged phase and fluorescence time-lapse images of WT and *pleC(ΔPAS)* cells with DivJ-eYFP (left). Arrowheads point to cytokinesis events. Arrows point to the appearance of a new DivJ-eYFP focus. Scale bar = 2 µm. Violin plot of Δ*t*, the time interval between cytokinesis and appearance of a new DivJ-eYFP focus (right). WT *n* = 413. *pleC(ΔPAS) n* = 471. Error bars denote mean ± standard deviation. An unpaired *t*-test was used to calculate *P* < 0.0001.

Western blots of synchronized cells showed that the master transcription factor, CtrA, and the chemoreceptor, McpA, were degraded more rapidly in *pleC(ΔPAS)* and *ΔpodJ* mutant cells than in WT cells (Fig. 5A and B). As a control, we probed the outer membrane protein, PAL, which does not change in abundance throughout the cell cycle (Fig. 5A) (40). It is possible that rapid degradation of CtrA and McpA in *ΔpodJ* might also be due to pleiotropic effects related to deletion of *podJ*. These results show that swarmer cell-specific proteins are degraded more rapidly in *pleC(ΔPAS)* and *ΔpodJ* cells, suggesting that these mutants have a shorter swarmer phase of the cell cycle compared to WT.

In a second assay to determine the timing of the swarmer to stalked cell differentiation, we measured the time between cytokinesis and initiation of chromosome replication. The ParB protein binds to the *parS* DNA sequence near the origin of replication, the first region of the chromosome that upon duplication is transported rapidly to the opposite cell pole (41). At the start of replication, the ParB protein bound to *parS* is located at the incipient stalked pole. Splitting of a single ParB focus into two distinct foci indicates duplication of the *parS* DNA sequence and the initiation of chromosome replication (41). Because initiation of chromosome replication occurs exclusively in stalked cells (39), this event can be used as a cell cycle landmark. Using time-lapse microscopy, we measured the time between cytokinesis and the splitting of the single eYFP-ParB focus into two distinct foci in the swarmer daughter (Fig. 5C). We found that this time interval was significantly shorter for *pleC(ΔPAS)* compared to WT with an average time difference of 5.9 ± 1.6 minutes (Fig. 5C). In addition, we observed that the ratio of cells with one chromosome compared to two chromosomes was lower in mixed populations of *pleC(ΔPAS)* and *ΔpodJ* cells compared to WT cells, consistent with there being fewer swarmer cells in these mutant strains (Fig. S5). These results suggest that initiation of chromosome replication occurs after a shorter swarmer cell duration in *pleC(ΔPAS)* cells compared to WT cells.

In a third assay, we measured the time difference between cytokinesis and the appearance of the DivJ histidine kinase which localizes to the stalked pole (6). During swarmer to stalked cell differentiation, swarmer pole proteins are degraded and newly synthesized stalked pole proteins, including DivJ, localize to the new stalked pole (4, 38). Accordingly, we measured the time interval between cytokinesis and the appearance of a DivJ-eYFP focus at the newly formed stalked pole. We found that this time interval was significantly shorter for *pleC(ΔPAS)* compared to WT with an average difference of 6.6 ± 1.4 minutes (Fig. 5D). Together these results suggest that *pleC(ΔPAS)* swarmer cells progress to stalked cells faster than WT swarmer cells and that PleC PAS domains play a critical role in regulating the duration of the swarmer phase of the cell cycle.

### PleC polar localization is not required for DivK dephosphorylation and pili biogenesis

**Fig 6:**
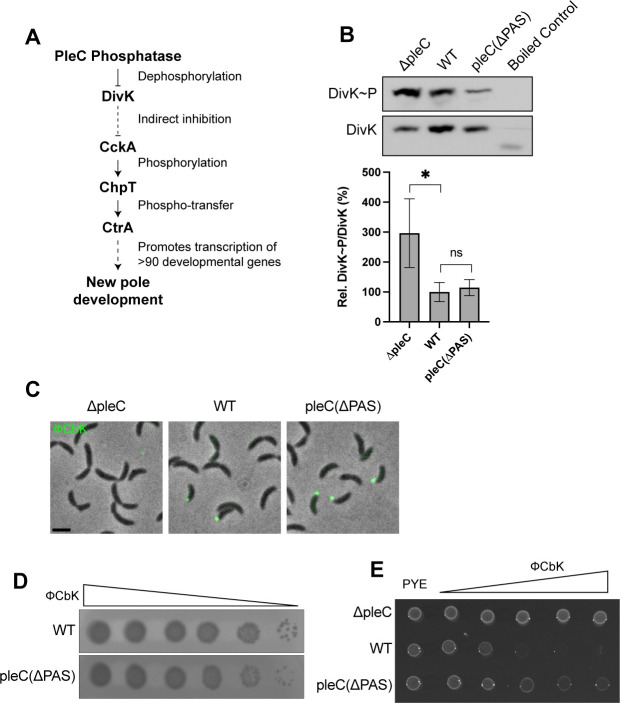
PleC polar localization is not required for DivK dephosphorylation and pili biogenesis. (A) Schematic of PleC phosphatase activity on DivK and the downstream molecular pathway that results in new pole development. (B) PhosTag SDS-PAGE immunoblots showing phosphorylated and unphosphorylated DivK-HaloTag-3xFlag using an anti-Flag antibody (top). Bar plot shows average ratios of phosphorylated to unphosphorylated protein as a percentage of the WT ratio (bottom). Separate exposure times were used to capture unsaturated phosphorylated and unphosphorylated bands. Signal density was averaged across three replicates. Error bars indicate standard deviation. An unpaired *t*-test was used to calculate *P* = 0.0457. (C) Fluorescent ϕCbK phage labeled overnight with 25 µM Sytox Green attaching to cells with polar pili. Scale bar = 2 µm. (D) Serial dilutions of ϕCbK spotted onto bacterial lawns that formed plaques overnight. (E) Cells grown overnight in the presence of serial dilutions of ϕCbK.

PleC phosphatase activity on DivK is required for downstream phosphorylation and activation of CtrA at the flagellar pole of pre-divisional cells (Fig. 6A). When DivK is phosphorylated, DivK~P forms a complex with the pseudokinase DivL and inhibits CckA kinase activity. Therefore, PleC phosphatase activity on DivK frees CckA from DivL-DivK~P inhibition and allows CckA kinase activity and phosphorylation of CtrA by phosphotransfer through ChpT (Fig. 6A) (8, 42, 43). When phosphorylated and active, CtrA promotes the transcription of over 90 genes that are involved in the development of the new flagellar pole, which includes flagellar rotation and pili biogenesis (Fig. 6A) (7, 44). To determine whether PleC phosphatase activity on DivK is impaired in *pleC(ΔPAS)* mutants, in which PleC is diffuse (Fig. 2), we performed PhosTag gel-electrophoresis to measure the ratio of DivK~P/DivK in cells expressing DivK-HaloTag-3xFlag as the sole copy of DivK from the endogenous locus. We found that the ratio of DivK~P/DivK was very high in *ΔpleC* (297% ± 115% of WT), as would be expected from removing a DivK phosphatase (Fig. 6B). The ratio of DivK~P/DivK in *pleC(ΔPAS)* (115% ± 27% of WT) was not significantly different from that of WT, suggesting that DivK dephosphorylation is not impaired in this mutant (Fig. 6B). We did not see a significant difference in the total levels of DivK protein in the different mutants (Fig. S3B).

It has been previously shown that PleC phosphatase and not kinase activity is required for the development of the new flagellar pole, including flagellar rotation and pili biogenesis (7). Because our results show that loss of PleC PAS domains does not impair PleC phosphatase activity, we suspected that new pole development would not be gravely impaired in *pleC(ΔPAS)* cells. To test this hypothesis, we assayed the ability of ϕCbK, a phage that infects *Caulobacter* through its polar pili, to attach to and infect *Caulobacter* cells (45, 46). We fluorescently labeled ϕCbK with Sytox Green, a membrane-impermeable DNA dye, and introduced labeled phage to *Caulobacter* cells. Phage attached to pili appeared as fluorescent puncta at the cell pole. We observed fluorescent ϕCbK puncta at the cell poles of WT and *pleC(ΔPAS)* swarmer cells indicating that these cells have polar pili (Fig. 6C). We did not see fluorescent ϕCbK puncta at the poles of *ΔpleC* cells which do not grow pili (Fig. 6C) (7). To assess whether cells were sensitive to phage infection, we spotted serial dilutions of ϕCbK onto bacterial lawns and observed plaque formation. Both WT and *pleC(ΔPAS)* cells formed plaques and are therefore susceptible to phage infection (Fig. 6D). We observed that the plaques at the lowest phage concentration were bigger in WT than in *pleC(ΔPAS)*, suggesting that this mutant might have slightly higher resistance to ϕCbK compared to WT. When cells were incubated with ϕCbK, and plated on PYE plates, we observed that *pleC(ΔPAS)* cells showed increased tolerance to high concentrations of phage compared to WT (Fig. 6E). This increased tolerance to ϕCbK is likely due to only swarmer and late pre-divisional cells having pili and being susceptible to ϕCbK. Because *pleC(ΔPAS)* spends less time in the swarmer period of the cell cycle, fewer susceptible swarmer cells are present in a mixed population. Together these results suggest that loss of PleC PAS domains does not impact PleC phosphatase activity and does not impair pili biogenesis at the new flagellar pole.

## DISCUSSION

### PleC phosphatase to kinase switch initiates swarmer to stalked cell differentiation

**Fig 7:**
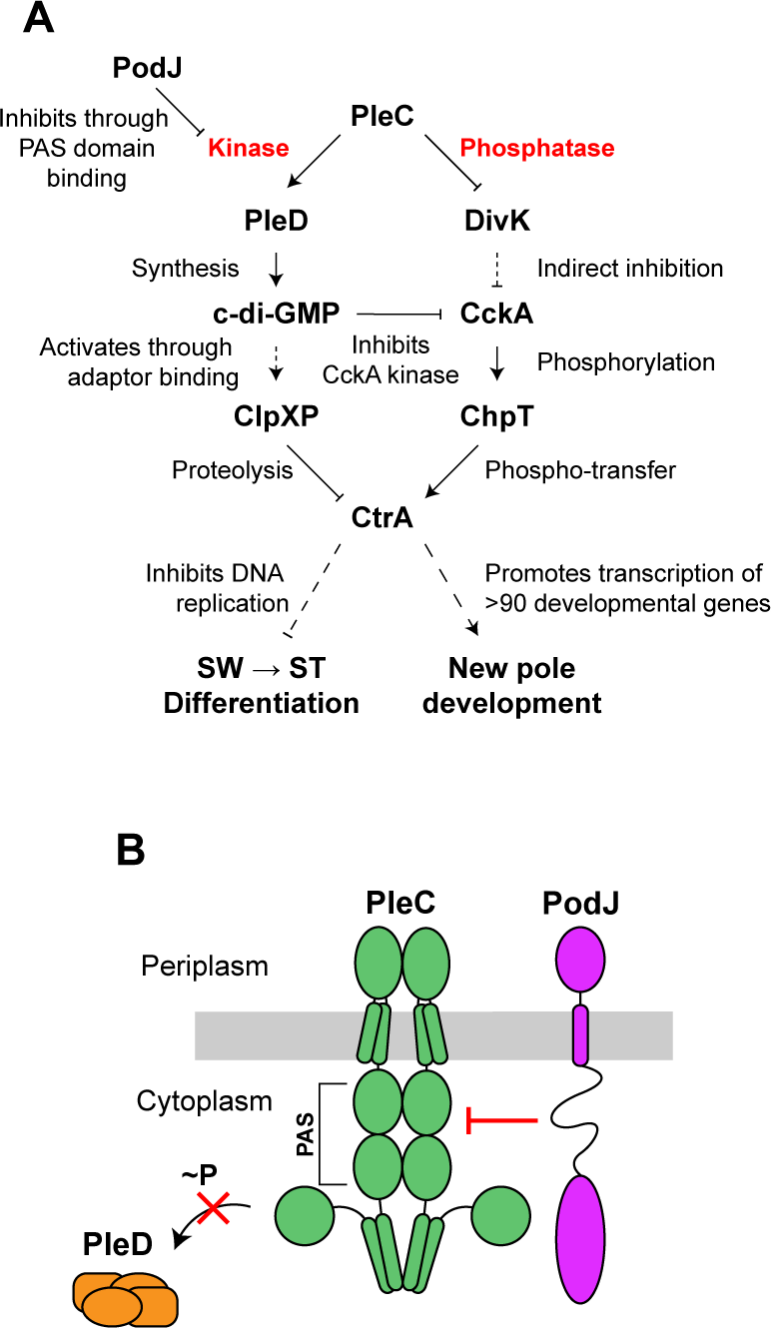
PodJ inhibition of PleC kinase activity controls timing of cell differentiation. (A) Schematic of molecular signaling pathways that result from PleC kinase and phosphatase activities and the downstream effects on swarmer (SW) to stalked (ST) cell differentiation and development of the new pole, respectively. (B) Model of PodJ interaction with PleC PAS domains, resulting in inhibition of PleC kinase activity on PleD.

PleC is essential for *Caulobacter* cell cycle progression, as it functions to phosphorylate PleD and to dephosphorylate DivK. The results that we have presented here, in conjunction with previously published data, suggest that PleC interaction with the scaffold protein PodJ at the cell pole inhibits PleC kinase activity on PleD, maintaining PleC in its phosphatase form during most of the cell cycle except for a narrow window of time in which PleC acts as a kinase following PodJ proteolysis (Fig. 1A and 7B). PodJ recruits PleC to the cell pole and inhibits PleC kinase activity on PleD through interaction with PleC’s PAS domains. PleC’s PAS domains have been shown to directly interact with the intrinsically disordered region (IDR) of PodJ *in vitro*, thereby inhibiting PleC autokinase activity (17). Our data support a model in which PodJ degradation releases PleC molecules from the cell pole. While the majority of these PleC molecules will undergo proteolysis (21, 22), a small fraction will switch to their kinase conformation and phosphorylate PleD. At this time, concurrent allosteric binding of DivK to PleC might further stimulate PleC kinase activity on PleD (5). Phosphorylated PleD synthesizes c-di-GMP, which binds to the ClpXP adaptor, PopA, enabling the degradation of the master cell cycle regulator, CtrA (Fig. 7A) (4). Proteolysis of CtrA is required for initiation of chromosome replication which occurs exclusively in stalked cells (39). Increased c-di-GMP concentration also activates ShkA which initiates stalked pole development (47). Therefore, PleC release from the cell pole and phosphorylation of PleD initiate a cascade of signaling events that results in swarmer to stalked cell differentiation. After differentiation, newly synthesized PodJ localizes to the incipient flagellar pole opposite the stalk, recruiting PleC to that pole, and reverting it back to its phosphatase form (Fig. 1A and 7B). As PleC is synthesized throughout the cell cycle (32, 48), newly synthesized PleC is also localized to the incipient flagellar pole at this time.

In prokaryotes, histidyl-aspartyl (His-Asp) signal transduction systems play a major role in cellular adaptation to environmental conditions. Most bacterial histidine kinases are bifunctional, having the ability to switch between kinase and phosphatase activities (49, 50). Some histidine kinases undergo conformational changes in response to environmental changes such as pH or osmolality, as in the cases of *Thermotoga maritima* HK853 or *Salmonella enterica* EnvZ (51, 52). Others change conformation through protein-protein interactions, such as PII-mediated phosphatase activation of NtrB (53). In *Caulobacter*, the histidine kinase, CckA, switches between kinase and phosphatase activity depending on its subcellular localization and interaction partners at the cell pole (54). Through reconstitution of CckA on liposomes, it has been shown that autokinase activity is stimulated by high concentrations of CckA (54). Therefore, at the flagellar pole, where CckA concentration is high, CckA autokinase activity is stimulated in a density-dependent manner. While the stalked pole also has a high CckA concentration, density-dependent autokinase activity is overshadowed by the inhibitory effects of direct binding of c-di-GMP to CckA, which has been shown to inhibit CckA kinase and promote its phosphatase activity (8, 43, 54, 55). There are many examples of histidine kinase crosstalk with non-cognate response regulators and histidine kinases with multiple cognate response regulators (56–58). PleC is an example of a histidine kinase that appears to have specified kinase and phosphatase activities toward its two substrates. In addition, because we observed similar ratios of PleD~P/PleD between WT and *ΔpleC* strains, PleC might also act as a phosphatase on PleD at an unknown point in the cell cycle (Fig. 3B). The signaling protein composition is distinctly different at the two *Caulobacter* poles, and this is the root basis of the asymmetric cell division that yields daughter cells of different cell fates (59, 60). Our results show that PleC’s interaction with PodJ at the new cell pole *in vivo* is the main determinant of its enzymatic state as a kinase or phosphatase. We speculate that other polar proteins such as DivL, which specifically localizes to the new cell pole and preferentially interacts with phosphorylated DivK (8), might reinforce specific kinase or phosphatase reactions on distinct substrates.

### Disruption of PodJ binding leads to PleC phosphatase to kinase switch

We show that upon deletion of PleC’s PAS domains, PodJ was unable to localize PleC to the pole and inhibit PleC kinase activity, resulting in premature swarmer to stalked cell differentiation and a shortened swarmer duration of the cell cycle (Fig. 2, 3B, and 5; Fig. S2). PodJ is sequentially degraded first by PerP cleavage of the PodJ periplasmic domain and then by MmpA which frees PodJ from the membrane, subsequently releasing PleC from the cell pole (18, 61). In addition to PodJ proteolysis, other factors may contribute to PleC release from the cell pole and switch to its kinase form. Tan et al. showed that the addition of purified SpmX protein to PodJ biomolecular condensates led to condensate disassembly and impaired recruitment of PodJ client proteins, including PleC (62). These results suggest that localization of newly synthesized SpmX to the incipient stalked pole might lead to dissociation of the PodJ polar complex and subsequent release of PleC from that pole. Medico et al. showed through bacterial-2-hybrid assays that PilA interacts with PleC’s N-terminal transmembrane domain and that *ΔpilA* swarmer cells take longer to synthesize c-di-GMP, suggesting a delayed swarmer to stalked cell differentiation in these cells. They proposed that upon pilus retraction, PilA enters the inner membrane and binds to and activates PleC kinase (10). This interaction with PilA might also play a role in PleC release from the cell pole and its switch to kinase activity.

### PAS domains allow modulation of enzymatic activity

PAS domains are found to integrate multiple cellular signals in different organisms (63). In this study, we showed that while PAS domains are required for PleC polar localization and inhibition of kinase activity on PleD, loss of PleC PAS domains does not affect PleC phosphatase activity on DivK, which is required for new pole development in pre-divisional cells (Fig. 2, 3B, and 6B; Fig. S2) (7). Mutant cells that have diffuse PleC(ΔPAS) as their only source of PleC retained their ability to grow pili and rotate their flagellum, suggesting that new pole development is not impaired in this mutant (Fig. 4B and 6B through E). Together these results suggest that signal integration through PleC’s PAS domains primarily leads to modulations in PleC kinase activity on PleD and not phosphatase activity on DivK.

We note that inhibition of kinase activity by PodJ might not be the sole function of the PleC PAS domains. DivK dephosphorylation by PleC located at the pole opposite of DivJ kinase activity on DivK has previously been suggested to contribute to an asymmetric distribution of DivK~P (7, 8). The spatial separation of DivK~P and DivK, together with the asymmetric distribution of c-di-GMP (64) and the CtrA~P gradient (65), allows for a robust integrated system that ensures proper differentiated cell fates of *Caulobacter* daughter cells. This redundancy seems to tolerate the diffuse localization of PleC(ΔPAS), but proper PleC localization might become essential for cell cycle progression in cells where CtrA~P or c-di-GMP spatial distribution is perturbed. We have shown that deleting PleC’s PAS domains leads to increased PleD phosphorylation in mixed populations, which presumably leads to a higher concentration of c-di-GMP in this mutant (Fig. 3B). However, further investigation is required to determine whether increased c-di-GMP in this mutant is cell cycle dependent. The effect of the PAS domains on PleC’s function reveals that cells can utilize a single histidine kinase to integrate cellular signals in a tailored response, allowing both phosphorylation and dephosphorylation of diverse targets, modulated by the presence or absence of context-dependent binding partners.

### Temporal regulation of cell differentiation as a possible means for adaptation

The timing of swarmer to stalked cell differentiation, occurred on average, approximately 6 minutes sooner for *pleC(ΔPAS)* cells compared to WT cells, during time-lapse microscopy experiments (Fig. 5C and D). While this difference in swarmer cell duration seems like a short amount of time, we anticipate that this small difference in time could result in major biological consequences for *Caulobacter* growing in the wild. *Caulobacter’s* di-morphic lifestyle allows it to disperse and colonize new habitats where it can form biofilms, which offer the advantage of increased tolerance to physical and chemical stressors. This di-morphic lifestyle enables *Caulobacter* to thrive in low nutrient environments such as lakes and streams (66). A swarmer duration that is 6 minutes shorter translates to a shorter distance that cells are able to disperse.

*Caulobacter* in the wild experiences environmental changes such as shifts in pH, temperature, and availability of nutrients. The ability of the species to utilize both lifestyles to its advantage hinges on *Caulobacter’s* ability to integrate signals from the environment into the decision of whether or not to differentiate. A previous study found that under conditions of carbon starvation, *Caulobacter* undergoes the initial morphological changes associated with stalked pole morphogenesis but stalls chromosome replication, not fully committing to the stalked cell fate (67). It has also been shown that the histidine kinase, DivJ, senses and responds to changes in ATP levels through its direct interaction with the SpmX biomolecular condensate. This study specifically showed that the SpmX IDR responds to changes in ATP levels, whereby low levels of ATP promote condensate formation, further stimulating DivJ kinase and preventing cell division defects associated with low DivJ kinase activity (59). As PleC PAS domains interact with PodJ’s IDR, it is tempting to consider that changes in the physical properties of PodJ’s IDR in response to environmental changes might also play a role in modulating PleC kinase activity, and ultimately regulate the timing of cell differentiation under specific environmental conditions.

The periplasmic Cache domain of PleC might also play a role in regulating PleC enzymatic activity. We show that deleting the periplasmic Cache domain led to reduced polar localization of PleC, suggesting that PleC interaction with a factor in the periplasm likely contributes to its polar localization (Fig. 2). Cache domains are sensory domains that typically bind to proteins or small molecules resulting in protein conformational changes that ultimately impact enzymatic activity (23). Changes in environmental conditions could result in Cache domain-mediated regulation of PleC kinase activity and therefore timing of cell differentiation.

The timing of cell differentiation is a critical factor in unicellular and multicellular development, which when mis-regulated can have severe biological consequences. Our analysis of the dynamic nature of PleC kinase and phosphatase activities, mediated by the interaction of the PleC PAS domains with the PodJ polar scaffold, enables the temporal regulation of cell differentiation. These results highlight the complexity of two-component systems and how bacterial kinases have evolved to integrate multiple signals into enzymatic activities functioning in time and space that result in distinct biological outcomes.

## MATERIALS AND METHODS

### Growth conditions

Unless otherwise stated, *Caulobacter* cells were grown from frozen stocks in PYE under aerating conditions at 30°C. Overnight PYE cultures were diluted accordingly into M2G or PYE in order to reach the desired OD600 the following day.

### Strain construction

All plasmids, bacterial strains, and primers used in this study are listed in Table S1. All strains are derivatives of LS101, a lab stock of NA1000/CB15N. The strain TNC41 was made by electroporation of pTC14 into LS101. The plasmid pTC14 was constructed by Gibson assembly of pYFPC-2 digested with NdeI and KpnI and PCR amplification of PleC(bp1545-2529) from NA1000 genomic DNA with primers TC15F and TC16R. The resulting plasmid was digested with NheI and SacI, HaloTag amplified with primers TC62F and TC63R was inserted by Gibson assembly. TNC104 and TNC372 were made by electroporation of pTC59 into LS101 and LS3778, respectively, followed by sucrose counter selection. The plasmid pTC59 was constructed by Gibson assembly of pNPTS138 digested with SpeI and EcoRI and PCR amplifications from NA1000 genomic DNA with primers (TC190F and TC191R) and (TC192F and TC193R). TNC260, TNC296, TNC298, TNC455, TNC456, and TNC458 were made by electroporation of pTC98, pTC147, pTC149, pTC259, pTC60, and pTC262 into TNC104, respectively. The plasmid pTC98 was constructed by Gibson assembly of pYFPC-2 digested with NdeI and KpnI and PCR amplification from NA1000 genomic DNA with primers TC303F and TC304R. The plasmid pTC147 was constructed by Synbio [pTC98 was digested with NdeI and BmgBI, and a synthesized DNA sequence containing ppleC:pleC(aa1-182)-*E. coli* ArcB(aa43-54)-pleC(aa277-393) was inserted]. The plasmid pTC149 was constructed by Gibson assembly of pYFPC-2 digested with NdeI and KpnI and PCR amplifications from NA1000 genomic DNA with primers (TC303F and TC85R) and (TC86F and TC16R). The plasmid pTC259 was constructed by Synbio (pTC98 was digested with SacI and NheI, and a synthesized DNA sequence encoding for 3xFlag was inserted). The plasmids pTC260 and pTC262 were constructed by Gibson assembly of pTC259 digested with NdeI and KpnI and PCR amplified regions from pTC147 and pTC149 with primers TC303F and TC304R. TNC506 and TNC507 were made by electroporation of pTC275 and pTC276 into TNC104, respectively, followed by sucrose counter selection. The plasmid pTC275 was constructed by Gibson assembly of pNPTS138 digested with SpeI and EcoRI with PCR amplifications from NA1000 genomic DNA with primers (TC411F and TC412R) and (TC528F and TC529R) and a DNA sequence synthesized by Integrated DNA Technologies (IDT) encoding 3xFlag with overlapping sequences. TNC511 and TNC512 were made by electroporation of pTC98 into TNC506 and TNC507, respectively. TNC515 and TNC518 were made by electroporation of pTC149 into TNC506 and TNC507, respectively. TNC533 and TNC711 were made by electroporation of pTC98 and pTC259 into TNC372, respectively. TNC670 was made by electroporation of pTC342 into LS101 followed by sucrose counter selection. The plasmid pTC342 was constructed by Gibson assembly of pNTPS138 digested with SpeI and EcoRI and PCR amplifications from NA1000 genomic DNA with primers (TC691F and TC85R) and (TC86F and TC692R). TNC688 and TNC689 were made by electroporation of pJP384 into LS101 and TNC670, respectively. TNC704 was made by electroporation of pTS18 into TNC670.

### PhosTag

Cells were grown overnight in M2G to an OD600 of 0.3–0.5. Samples were normalized to an equivalent of 1 mL of cells at OD600 of 0.4, and cell pellets were flash frozen in liquid nitrogen and stored at −80°C. Within 24 hours of flash freezing, samples were lysed at room temperature for 5 minutes in 75 µL of 10 mM Tris-HCl (pH = 7), 4% SDS, 2 µL DNase, supplemented with PhosStop phosphatase inhibitor cocktail tablet (Roche 4906837001), and then placed directly on ice. A sample of WT cells was boiled at 95°C for 5 minutes to serve as a negative control corresponding to the unphosphorylated band. Samples were then centrifuged for 5 minutes at 13,000 RPM, and 12 µL of the supernatant was then added to 12 µL of Bio-Rad 2× Laemmli sample buffer with 5% β-mercaptoethanol. From these samples, 20 µL was loaded onto PhosTag gels. PhosTag poly-acrylamide gels were made with a final concentration of 100 mM ZnCl_2_ as described by the Wako PhosTag guidebook. Phosphorylated and unphosphorylated bands were separated by gel electrophoresis at 4°C. Gels were washed three times in Towbin buffer containing 10 mM EDTA for 10 minutes at room temperature followed by one wash without EDTA. Protein was transferred onto Polyvinylidene fluoride (PVDF) membranes by semi-dry transfer for 4 hours followed by standard Western blot antibody probing procedures.

### Western blot

Cells grown overnight in M2G were normalized to an equivalent of 1 mL of cells at an OD600 of 0.4, and pellets were either flash frozen in liquid nitrogen and stored at −80°C or immediately lysed. To lyse, cells were resuspended in 40 µL dH_2_O and combined with 40 µL of Bio-Rad 2× Laemmli sample buffer with 5% β-mercaptoethanol and boiled at 95°C for 10 minutes. Samples were loaded onto precast Bio-Rad 4%–15% gradient poly-acrylamide gels followed by gel electrophoresis. Protein was transferred to PVDF membranes by semi-dry transfer for 2 hours. Sigma monoclonal anti-Flag M2 antibody (F1804) (1:4,000 dilution) or rabbit polyclonal anti-serra recognizing CtrA (1:10,000 dilution), McpA (1:20,000), or PAL (1:50,000 dilution) were used for immunoblots, followed by Abcam goat-anti-mouse HRP (ab205719) (1:10,000 dilution) or Abcam goat-anti-rabbit HRP (ab97051) (1:10,000 dilution). SuperSignal West Pico PLUS or SuperSignal West Femto Chemiluminescent Substrate from Thermo Scientific was used to detect Horseradish peroxidase (HRP). Density measurements were calculated using Li-Cor Image Studio Lite. For CtrA synchrony signal density measurements, WT and *pleC(ΔPAS)* samples were run on the same gel.

### Synchrony

Cells were grown overnight in M2G to an OD600 of 0.4–0.5, 1 L for large-scale or 40 mL for small-scale synchronies. Cells were pelleted and washed with cold M2. Cells were resuspended in 1:1 M2 to Percoll, and swarmer cells were separated from stalked and pre-divisional cells by density centrifugation (large scale: 1 hour at 6,000 RPM, small scale: 20 minutes at 11,000 RPM) at 4°C. The higher band consisting of stalks and pre-divisional cells was aspirated, and swarmer cells were washed in cold M2 before being released into M2G and grown at 30°C. At appropriate time points, cells were removed and normalized to an OD600 of 0.4 and flash frozen in liquid nitrogen and stored at −80°C.

### Imaging

Unless otherwise stated, all cell imaging was done on M2G 1.5% agarose pads on a light-emitting diode-based (Lumencor, SpectraX) multicolor epifluorescence microscope consisting of a Leica Dmi8 stand equipped with an immersion oil phase contrast objective (100×, HC PL APO, 1.4 numerical aperture) and an EMCCD camera (Hamamatsu, C9100 02 Cl). Cells were grown overnight in M2G to an OD600 of 0.3–0.4. Images were analyzed and processed using Fiji software (68). For generating line profiles, the Fiji plugin, MicrobeJ (69), was used to divide cells into either 26 or 50 bins along the longitudinal axis, and the average fluorescence intensity of each bin was used to generate fluorescence profile plots. The protocol for imaging ϕCbK attachment was adapted from Hinz et al. (15). Briefly, Sytox Green (ThermoFisher S7020) was added to 1 mL of ϕCbK to a final concentration of 25 µM and allowed to incubate overnight at 4°C. Cells were mixed 1:1 with fluorescently labeled phage and imaged.

### Time-lapse imaging

For time-lapse imaging, swarmer cells isolated from a small-scale synchrony were either prepared for imaging immediately or allowed to grow to the appropriate stage of the cell cycle prior to imaging. For HaloTag imaging, cells were incubated in 2 nM Janelia Fluor 549 dye (Promega GA1110) in M2G for 10 minutes at room temperature and washed three times with M2G prior to imaging. Images were taken at 10-minute time intervals. For PleC-HaloTag, cells were labeled immediately after synchrony. For DivJ-HaloTag, swarmer cells were allowed to progress to stalked cells for 40 minutes prior to labeling. For imaging DivJ-eYFP and eYFP-ParB cells, isolated swarmer cells were grown in M2G or M2G with 0.01% xylose for 1 hour to allow cells to progress to the pre-divisional stage. Agarose pads made with M2G or M2G with 0.01% xylose were cut in half in order to image WT and *pleC(ΔPAS)* cells simultaneously. The microscope was programmed to take images at 5-minute intervals and move the stage to take images of both halves of the agarose pad. The genotypes associated with raw images were blinded, and Δ*t* intervals were acquired manually.

### Single cell tracking

For single cell tracking, Nunc Lab-Tek II Chambered Coverglass (ThermoFisher 155360) glass bottom wells were treated with 1 M KOH for 30 minutes and then washed three times with dH_2_O. Swarmer cells isolated from a small-scale synchrony were resuspended in 50–200 µL of cold M2. Prior to imaging, 10 µL of cells in cold M2 was added to 190 µL of room temperature M2G with 20% glycerol and allowed to assimilate for at least 3 minutes. Images were taken at a focal plane above the coverslip in order to not capture cells that had settled to the bottom of the well. Images were captured at 52 ms intervals. The Fiji plugin, TrackMate, was used to track individual cells and calculate speeds (36).

### Flow cytometry

For flow cytometry experiments, cells were grown overnight in M2G to an OD600 of 0.4–0.5. Rifampicin was added to cell cultures to a final concentration of 15 mg/mL and allowed to grow for an additional 3 hours to prevent new rounds of chromosome replication. Cells were incubated overnight at 4°C in 70% ethanol in M2. The following day, cells were resuspended in M2 containing a final concentration of 5 µM of Sytox Green (ThermoFisher S7020). Samples were run on an Agilent NovoCyte Penteon Flow Cytometer. The software FlowJo was used to analyze data and generate histograms.

### Plate assays

For all plate assays, images were taken with a Bio-Rad ChemiDoc MP Imager. For swarm assays, single colonies were poked into PYE 0.3% agar plates and allowed to grow at 30°C for 3 days prior to imaging. Swarm areas were calculated using Matlab and averaged over replicate plates. For plaque assays, 400 µL of mid-log cells was combined with 4 mL melted PYE with 0.3% agar and poured on top of regular PYE plates (1.5% agar) and cooled to room temperature. 1.5 µL of 10× serial dilutions of ϕCbK phage was spotted onto plates. Plates were incubated at room temperature overnight, and images were taken the next day. To assay for cell growth, 1.5 µL of 1:1 mixtures of mid-log cells with 10× serial dilutions of ϕCbK was spotted onto PYE agar plates. Plates were incubated at room temperature overnight, and images were taken the following day.

## Data Availability

All graphs and plots were made using either Prism or Matlab software. All codes generated by this study are available through Zenodo.

## SUPPLEMENTAL MATERIAL

Fig. S1 mbio.02125-23-s0001.tif PleC mutant constructs have stable *in vivo* expression.

Fig. S2 mbio.02125-23-s0002.tif PodJ is required for PleC polar localization.

Fig. S3 mbio.02125-23-s0003.tif Total levels of PleD and DivK are similar between *pleC* mutants.

Fig. S4 mbio.02125-23-s0004.tif PleD protein levels are greatly reduced in *podJ* mutant.

Fig. S5 mbio.02125-23-s0005.tif *pleC* and *podJ* mutations result in fewer G1-phase cells.

Table S1 mbio.02125-23-s0006.xlsx Bacterial strains, plasmids, and primers.

ASM does not own the copyrights to Supplemental Material that may be linked to, or accessed through, an article. The authors have granted ASM a non-exclusive, world-wide license to publish the Supplemental Material files. Please contact the corresponding author directly for reuse.
