# Supplementary figures and images for: Phosphatase to kinase switch of a critical enzyme contributes to timing of cell differentiation
Source: mBio. 2023 Dec 6;15(1):e02125-23. doi: 10.1128/mbio.02125-23 (PMC10790692; doi:10.1128/mbio.02125-23)

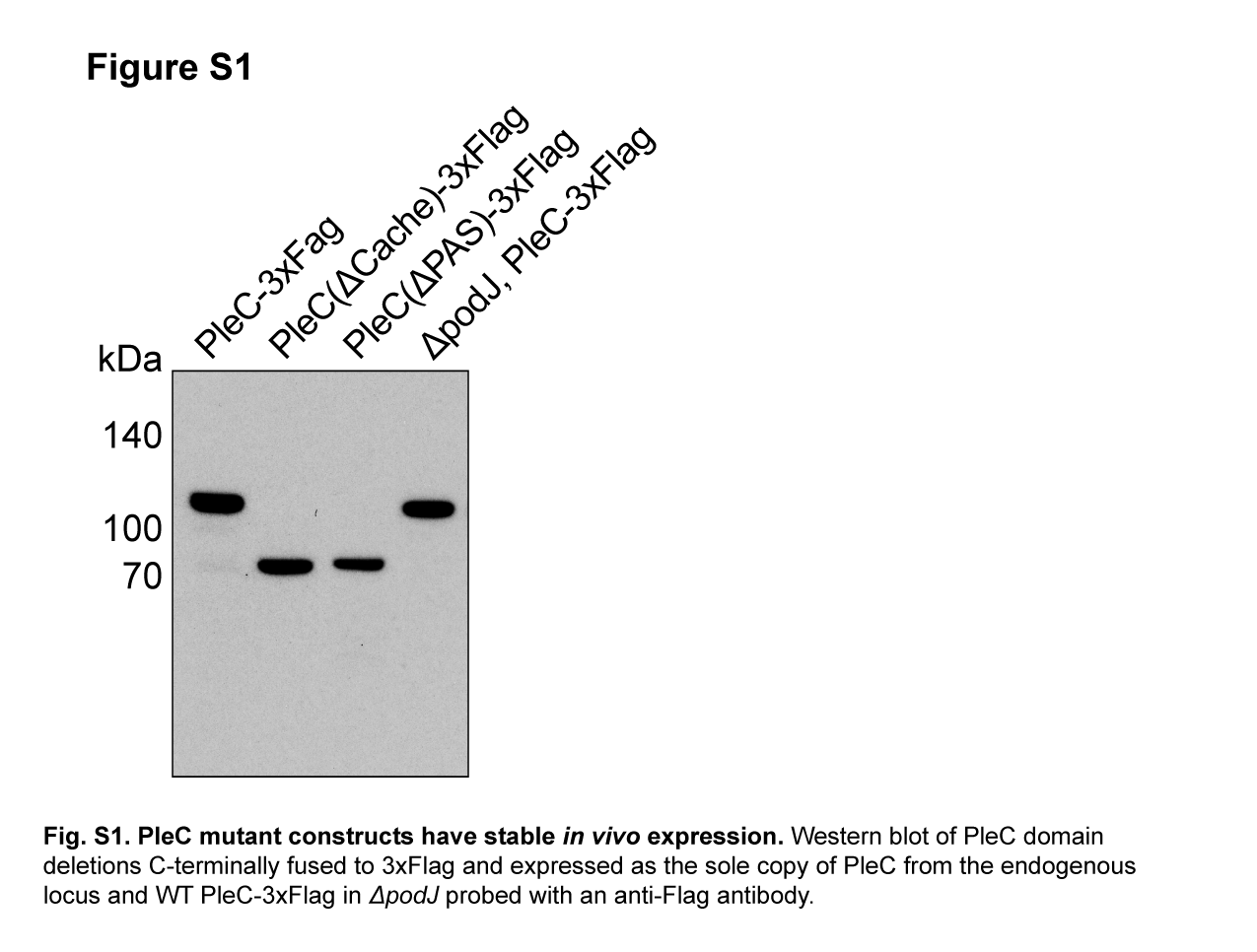

Supplement: Fig. S1 — PleC mutant constructs have stable in vivo expression. [file mbio.02125-23-s0001.tif]

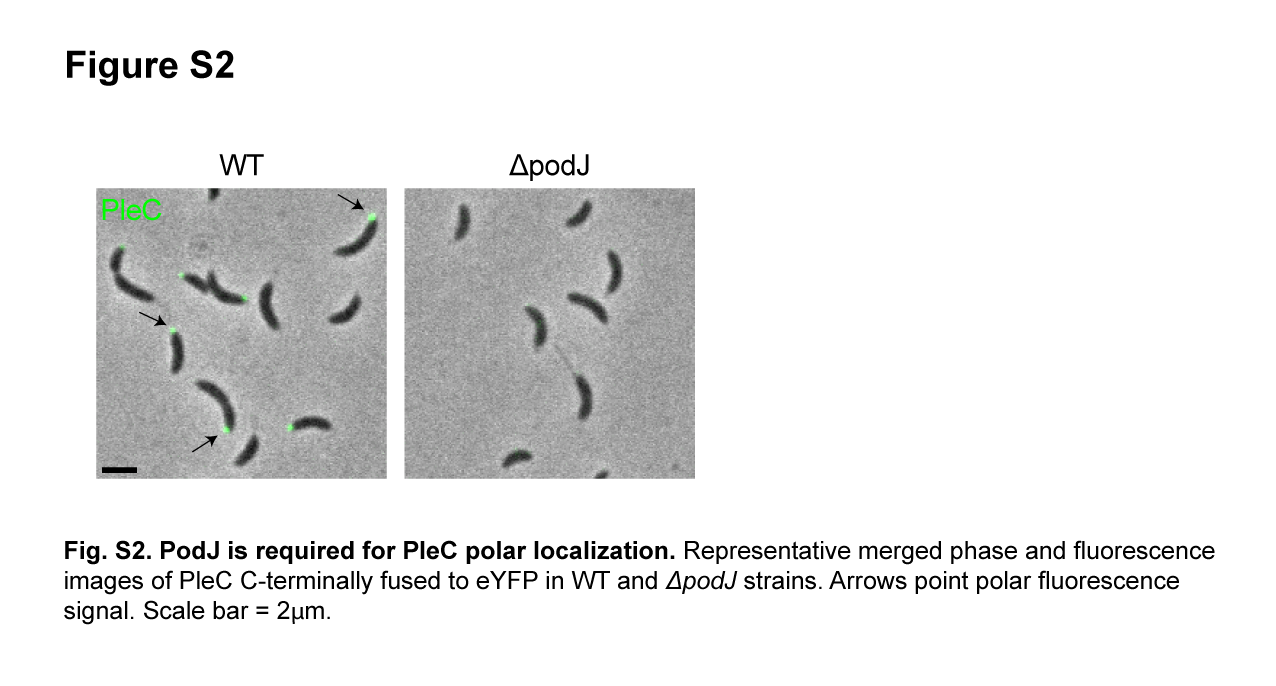

Supplement: Fig. S2 — PodJ is required for PleC polar localization. [file mbio.02125-23-s0002.tif]

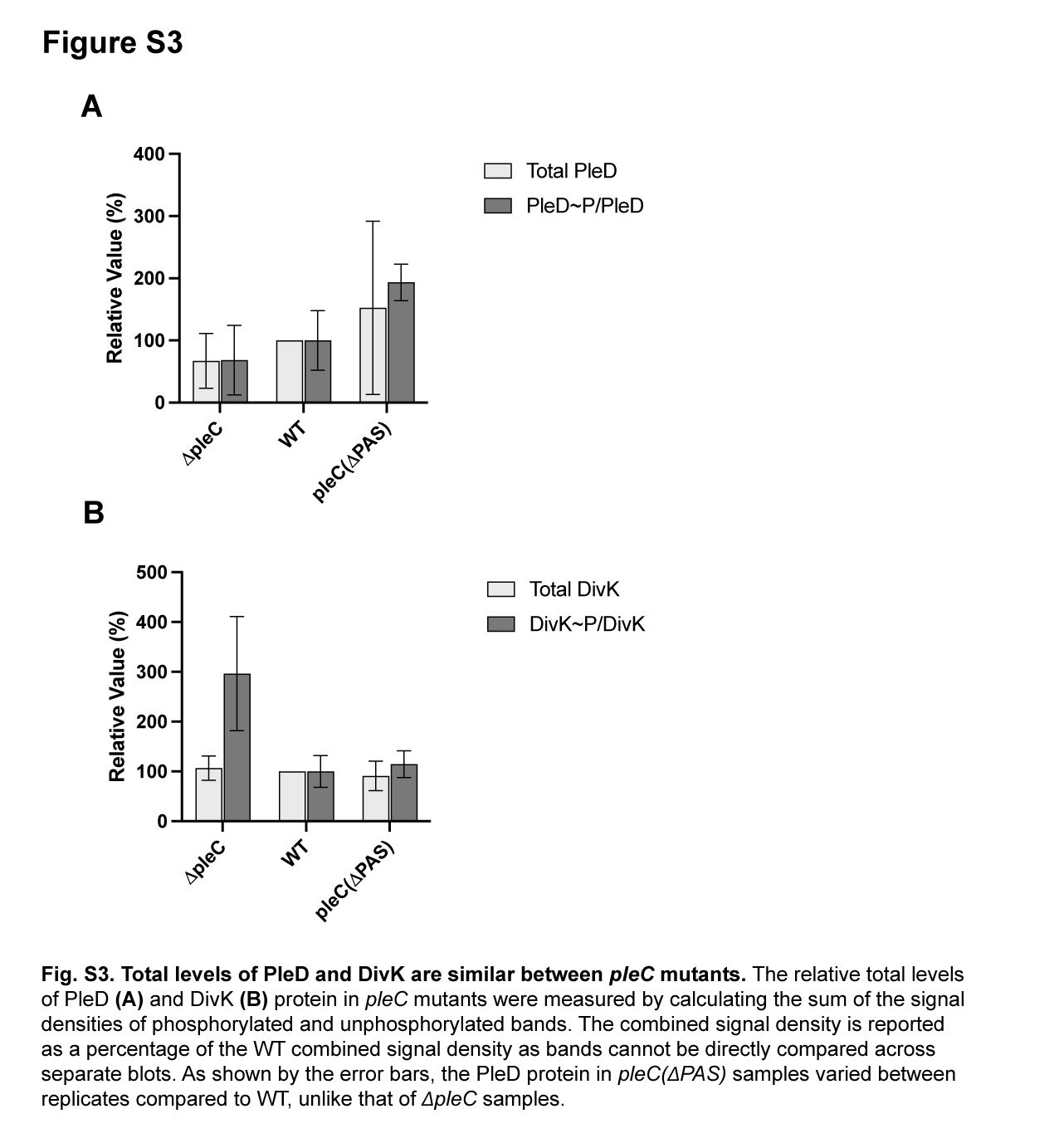

Supplement: Fig. S3 — Total levels of PleD and DivK are similar between pleC mutants. [file mbio.02125-23-s0003.tif]

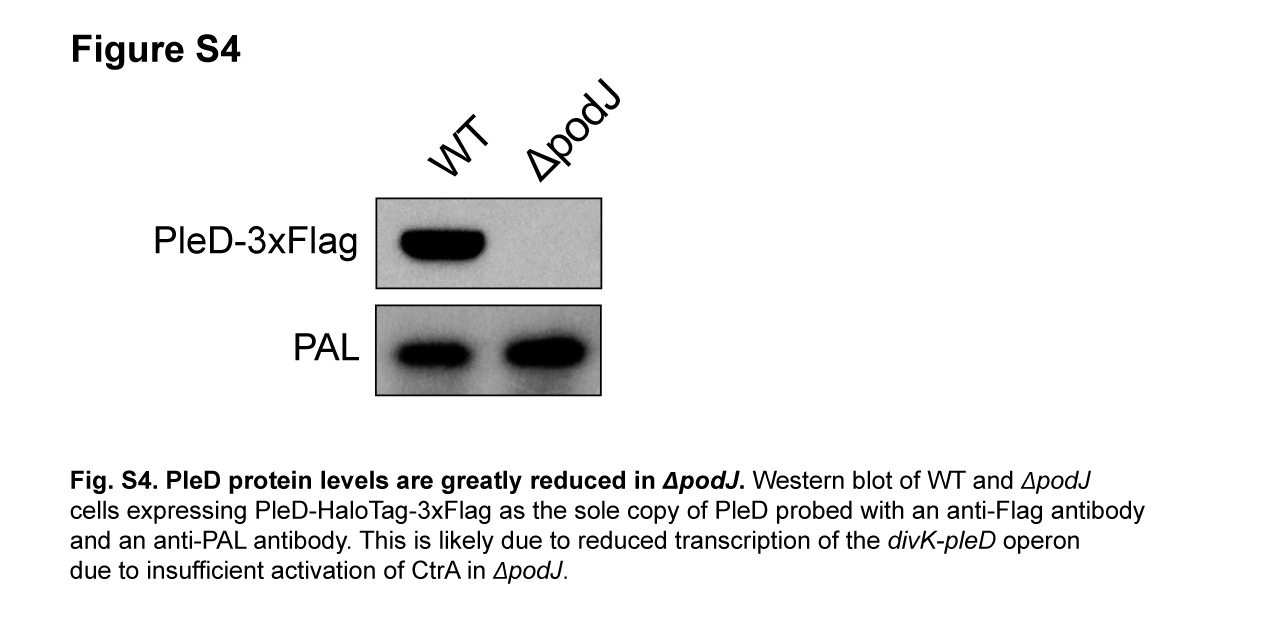

Supplement: Fig. S4 — PleD protein levels are greatly reduced in podJ mutant. [file mbio.02125-23-s0004.tif]

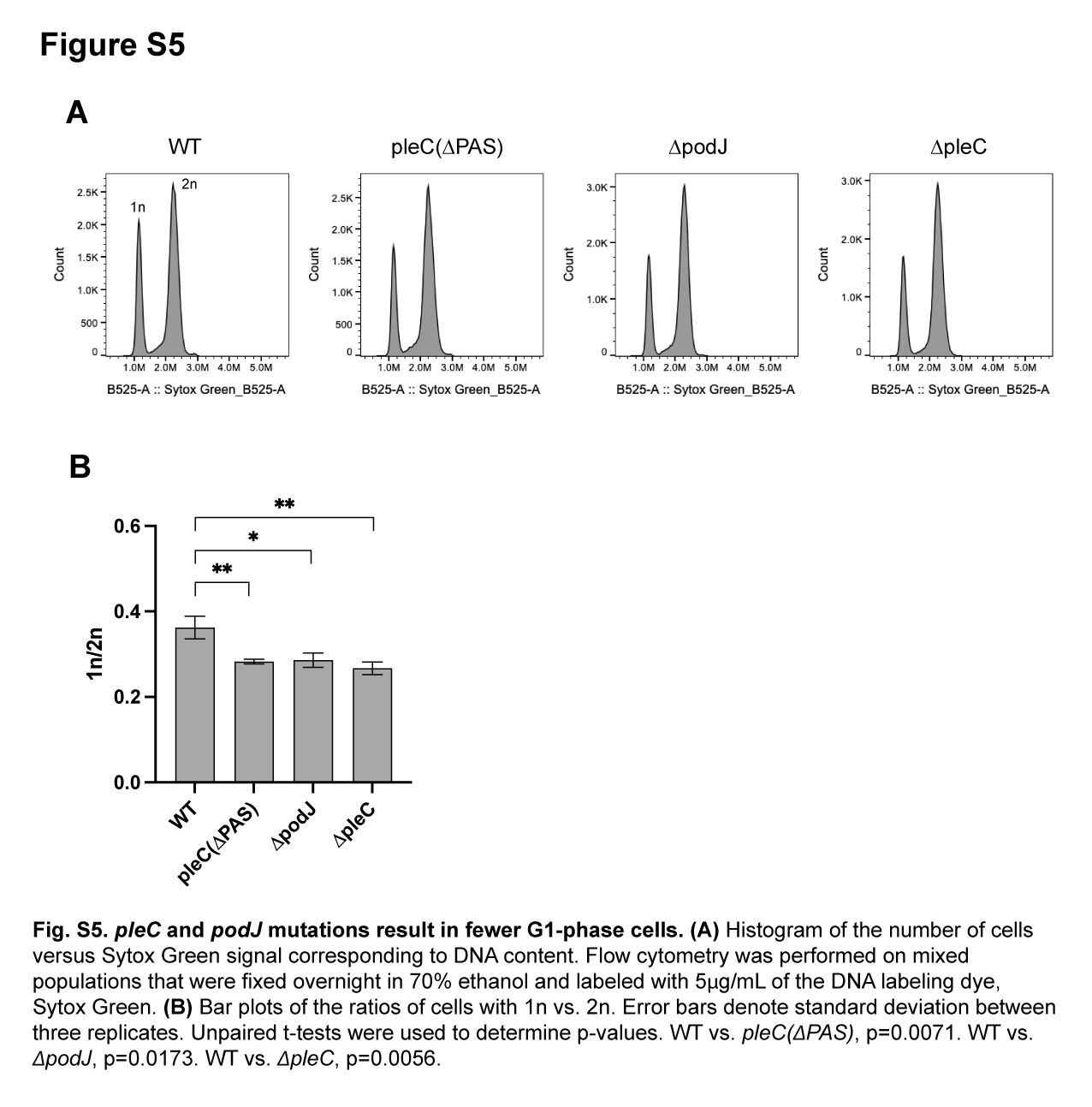

Supplement: Fig. S5 — pleC and podJ mutations result in fewer G1-phase cells. [file mbio.02125-23-s0005.tif]
